# Comparing driving behaviors and physiological features of older adult drivers with and without elevated amyloid burden

**DOI:** 10.1002/alz.089613

**Published:** 2025-01-03

**Authors:** Anu M Kumar, Yi Lu Murphy, Amanda Cook Maher, Carol C Persad, Emma Flynn, Jordan R Bross, Robert A. Koeppe, Bruno Giordani

**Affiliations:** ^1^ University of Michigan, Ann Arbor, MI USA; ^2^ University of Michigan ‐ Dearborn, Dearborn, MI USA; ^3^ University of Michigan Medical School, Ann Arbor, MI USA

## Abstract

**Background:**

Identifying markers for increased risk of cognitive change and early Alzheimer’s disease (AD) is key to enhancing the effectiveness of treatments. Changes in instrumental activities of daily living, including the complex task of driving, may serve as potential markers to identify older adults at risk of cognitive decline. This study evaluates driving behaviors and associated physiological signals in older adults with and without elevated brain amyloid burden.

**Method:**

Video and physiological data from 21 amyloid positive and 21 amyloid negative consensus diagnosed cognitively normal participants over the age of 65 who participated in the University of Michigan’s Driving and Physiological Responses study were analyzed. Amyloid positivity was determined based on the PET centiloid scale. All drivers completed the same fixed course route. Road‐ and driver‐view videos of each participant were annotated to mark 40 key road events and driving behaviors, such as right turns and looking at the rear‐view mirror. Heart rate (HR) and electrodermal activity (EDA) data collected through an Empatica E4 watch were analyzed for each road/driving event (e.g., average HR during freeway entrance). Independent sample t‐tests with α’s = 0.05 were used to look for differences between amyloid positive and negative groups.

**Result:**

HR responses to intersections without signs and freeway entrance ramps were consistently higher among amyloid positive drivers compared to amyloid negative drivers (*p*’s<0.05). Furthermore, amyloid positive drivers showed a pattern of lower EDA responses to freeway exit ramps than amyloid negative drivers (*p*<0.05).

**Conclusion:**

Cognitively normal amyloid positive older drivers demonstrated consistently higher HR and lower EDA in key driving situations compared to their amyloid negative peers. Findings suggest physiological responses of older drivers under different driving conditions may represent markers for identifying those individuals at higher risk of future cognitive decline. These results can be used to build an accurate and generalizable Machine Learning‐based AD diagnostic tool to detect early‐stage AD in drivers.

